# Culturally appropriate care to support maternal positions during the second stage of labour: Midwives’ perspectives in South Africa

**DOI:** 10.4102/phcfm.v14i1.3292

**Published:** 2022-04-25

**Authors:** Maurine R. Musie, Mmapheko D. Peu, Varshika Bhana-Pema

**Affiliations:** 1 Department of Nursing Science, Faculty of Health Sciences, University of Pretoria, Pretoria, South Africa; 2 Department of Advanced Nursing Science, Faculty of Health Sciences, University of Pretoria, Pretoria, South Africa

**Keywords:** culturally appropriate care, birth choices, birth position, maternal positions, midwives, second stage of labour

## Abstract

**Background:**

‘Doing what the Romans do in Rome’ was an expression raised by one of the midwives following workplace culture and disregarding women’s birth choices. Midwifery practice in South Africa caters for a culturally diverse ethnic groups of childbearing women. Culturally appropriate care highlights the importance of including women in decision-making concerning their birth preferences including maternal positions during labour. Women’s right to choose their maternal position and cultural preferences during labour has been overlooked, leading to poor maternal healthcare provision and negative birth experiences.

**Aim:**

In this article, the researchers aimed to describe and explore midwives’ perspectives on culturally appropriate care to support maternal positions during the second stage of labour.

**Setting:**

Midwives working in the maternity ward in a public hospital in South Africa.

**Methods:**

A qualitative descriptive design using individual interviews was used to collect data. The participants were selected using the purposive sampling method. The study population comprised 20 midwives who volunteered to participate in the study. Data were transcribed manually and analysed using thematic analysis.

**Results:**

The four main themes are as follows: (1) Caring for women from various ethnic groups, (2) midwives disregard women’s beliefs and culture, (3) midwife personal cultural attributes and (4) midwifery unit workplace culture.

**Conclusion:**

The authors concluded that culturally appropriate care towards the women’s choices of birth position during the second stage of labour should form an integral part of the midwifery care rendered.

## Introduction

The need for ‘culturally appropriate’ maternity care services is core to the World Health Organization’s (WHO) strategy for improving maternal and new-born health and ending preventable maternal mortality.^1,2^ Health professionals (including midwives) are expected to provide culturally appropriate care to childbearing women.^3^ Culturally appropriate care includes, among others, the involvement of the labouring women in the decision-making regarding preferred birth choices and maternal birth position during labour.^4^ Moreover, other studies reported culturally appropriate care during labour that includes allowing women the comfort and support from a birth companion of choice.^5^ This inclusion of women in decision-making is noted as one of the key indicators of culturally appropriate care that fosters quality care and long-lasting relationships between a woman and the healthcare professionals including midwives as embedded in the respectful maternity care (RMC) principles.^1,6^

Minority ethno-linguistic or religious childbearing women often have limited access to maternity care services than the rest of the population.^1^ This is very true in the South African context as well, ‘Midwives attend to women from various ethnic and religious backgrounds during intrapartum care’. This is due to migration and globalisation in the country that led to diversity and a *global village* of maternity care facilities that have been utilised by women from indigenous populations from several African countries (such as Zimbabwe, Ethiopia, Somalia and Nigeria) who have their own cultural preferences and practices during birth pertaining to birth position during the second stage of labour. A study conducted in Zimbabwe indicates that most childbearing women perceive the health facilities as foreign environments that were not comforting as traditional and cultural sources of care.^7,8^ In a country like Uganda, cultural customs like the use of herbs, a non-supine birth position and placental disposal are regarded as some of the sacred cultural preferences held by women.^9^ A systematic review conducted in various sub-Saharan African countries (Ethiopia, Kenya, Ghana and Nigeria) indicate that most women prefer to adopt maternal upright positions such as kneeling and squatting, which are guided by their instincts and cultural norms rather than being restricted on supine lithotomy positions during birth.^7^ The lack of women’s choice of birth position led to women preferring to have home births conducted by a traditional midwife and traditional birth attendant (TBA) rather than facility births with midwives, as facility care is not tailored towards the childbearing women’s cultural preferences and norms.^7^ Similarly, in Australia, there have been reports of disparities in terms of culture and cultural practices that exist between nurses and childbearing women. As a result, this has affected women’s utilisation of formal healthcare services.^10^ Thus, it is mandatory for skilled birth attendants (SBAs) (including midwives) to respect the cultures, values and beliefs of women.^10^

According to Mselle,^11^ the childbearing women’s choice of birthing positions is influenced by women’s empowerment and involvement in the birth process, age, parity, culture, the biomedical model and the birth attendants who are primary caregivers during the labour process. Presently, there is an outcry by some of the women from Africa (in countries such as Tanzania), complaining that the childbirth facilities are not culturally appropriate towards the women’s choices and are often termed as dehumanising.^11^ A systematic review, done by Jones, found that the lack of culturally appropriate care in maternal health can impede women’s utilisation of skilled birth care.^1^ In the context of the study, the authors focus on women’s cultural beliefs and practice regarding maternal positions adopted during the second stage of labour. The second stage of labour starts when the cervix reaches full dilatation (10 cm) and ends with the delivery of the baby.^12^ The maternal guidelines in South Africa indicate that a woman should be given a freedom of choice to mobilise and adopt upright maternal positions that come naturally to her during birth.^12^ Several advantages have been associated with the other upright birth positions that include more improved maternal outcome of the good progress of labour as a result of adequate labour contractions, increased pelvic outlet, adequate foeto-placental perfusion and reduced perineal tears.^13^ However, this is not a reality in South Africa where women are restricted on lithotomy/supine position.

The right of choice of birth position, freedom of movement during birth and continuous support during the first and second stage of labour has been overlooked in various midwifery settings.^14^ Approximately one in five births, which is approximately 22%, took place outside the healthcare facility globally.^15^ A study conducted in Northern Ghana further indicated that about 60% of women in Africa prefer homebirths assisted by TBA rather than facility birth with the midwives. The current healthcare system can be presumed to lack culturally appropriate care interventions, as the right of women to make an informed decision regarding birth position is often overridden and that can be seen as mistreatment during birth and labour.^16^ Women from various ethnic groups are assisted during antenatal, intrapartum and postpartum care at the health facilities. These women have certain cultural preferences regarding their birth process, which is often overlooked. Women are not included in the decision-making regarding their cultural preferences in relation to birthing positions. This lack of involvement of women is associated with negative birthing experiences.^16^ The midwives continue to position a significant majority of 68% women to give birth lying on their back in contradiction to their own choice of birth position.^3^ Furthermore, evidence-based practices for intrapartum care and midwifery care guidelines^12^ stipulate ‘that women should not be restricted to supine position during second stage of labour’ as this practice is associated with negative maternal and neonatal outcomes. Some of the negative maternal outcomes reported include prolonged labour, increased perineal trauma, and postpartum haemorrhage.^17^ Postpartum haemorrhage (PPH) accounts for 22% of maternal mortality and morbidity for every 100 000 live births.^18^ Postpartum haemorrhage is one of the big five causes of maternal mortality in South Africa and accounts for one-third of maternal deaths in Africa.^18^ Consequently, health professionals continue to turn a blind eye and continue to routinely place women on lithotomy position and restrict the women’s movement during the second stage of labour.

‘Lack of culturally appropriate care and intervention to the women’s needs is a global concern and has been noted as barrier’.^1,2^ Culturally appropriate care includes the simplest things, such as the midwives respecting women’s home language; however, in this study, it is clear that midwives found that the ethno-linguistic aspect was a barrier to provide culturally appropriate care, which is not in line with the WHO recommendations. The WHO vouch for ‘culturally appropriate’ maternity care services to improve maternal and neonatal outcomes, which later informs RMC.^1^ Most recently, the WHO made *recommendations on intrapartum care for a positive childbirth experience,* which alludes that it is mandatory that midwives should provide RMC.^4^ Respectful maternity care refers to midwives, taking the authority and responsibility to care for the women in a way that their rights, dignity and choices are respected. One of the fundamental rights of a woman include respect of her religion, beliefs and culture regarding choices during labour.^12^ We also trace back since time immemorial that birth positions could be freely changed and modified according to the parturient’s desires. Previous research studies indicate the history of birth position in Africa. It is evident that women were giving birth in various alternative birth positions, such as sitting, upright position, squatting, kneeling and using hands and knees and the left lateral birth positions.^19^ Furthermore, it is noted that the use of these alternative birth positions gives comfort to the women and is associated with better outcomes during birth. For changes to occur within the healthcare system and from the individual point of view, there is a need to look at the practices currently done within the maternity units, in order to assess whether they incorporate culturally appropriate care. The aim of this study was to explore and describe midwives’ perspectives on culturally appropriate care to support maternal positions during the second stage of labour.

## Methods

### Study design

A qualitative, exploratory and descriptive research design was used to explore and describe the culturally appropriate care to support women’s choice of maternal position during labour. This approach enabled the researchers to gain more in-depth understanding of the phenomenon studied.

### Setting

The study was conducted in a public hospital premises situated in Pretoria in the City of Tshwane Metropolitan district. The selected hospital operates 24 hours a day, and the services provided are low-risk maternal healthcare services that include antenatal, intrapartum and postpartum care. Overall, the hospital caters for rural and urban areas surrounding the hospital.

The study took place in the maternity ward of the hospital. This is a 30-bedded ward with eight ante-natal beds in one cubicle (two admission beds, two isolation, two first-stage and four second-stage/delivery beds located in private individual rooms). The labour ward offers services to low-risk childbearing women, without any obstetrical complication or high-risk conditions (such as pre-eclampsia). On average, about 300–350 women give birth monthly in the maternity ward.

The study population includes midwives (registered with the South African Nursing Council [SANC]) offering maternal health services to women from various ethnic backgrounds such as Tshwana speaking, Shona, Indian, Somalian, Venda, Zimbabwean, among others. These women come to the labour units for intrapartum care, and they have cultural practices and traditions that they follow (e.g. Muslims prefer consuming halaal food and some herbal drinks for protection during labour, women from African countries prefer squatting during labour, etc). Other low-risk childbearing women come as referrals from local clinics within the sub-district of Tshwane, and some are referrals from the nearby high-risk hospitals. The staff ratio is about eight midwives during the day and night shift who are present in the ward, which is in line with the SANC regulation.

### Population and sampling strategy

The researchers were working at the labour ward during the time of data collection in 2018. Permission was obtained from the hospital authorities as part of the recruitment strategy. The researchers utilised purposeful sampling to select the participants. The population of the study comprised 20 midwives who met the inclusion criteria and agreed to participate. All the midwives selected for the study were registered with SANC per the inclusion criteria (refer to Table 1 for demographic information on the participants).

**TABLE 1:** Demographic characteristics of the participants.

Variable	*n*	%
**Professional designation**		
Advanced midwife	7	35.00
Professional nurse	13	65.00
Community service	2	10.00
**Age profile (years)**		
20–29	7	35.00
30–39	7	35.00
40–49	3	5.00
50–59	2	10.00
60–69	1	0.05
**Gender**		
Female	20	100.00
**Years of experience**		
< 1	2	10.00
1–5	9	45.00
6–10	4	20.00
11–20	2	10.00

The identified midwives were then approached for participation in the study. Explanation was given to the participants regarding the nature of the study. Those who showed interest to participate in the study were also informed that their participation is voluntary. The characteristics of the population in this study were professional nurses with midwifery qualifications integrated during the comprehensive nursing degree/diploma training or 3-year diploma course and advanced midwives with a speciality in midwifery. The midwives were responsible for assisting women during the first and second stages of labour.

### Data collection

The study was conducted over four months during June–September 2018. Individual semi-structured, face-to-face interviews were employed to explore the midwives’ perspectives on culturally appropriate care to support maternal positions during the second stage of labour. First, the researchers scheduled suitable interview dates with the midwives for data collection. The interview took place in a quiet private room in the labour ward without disrupting the daily routine of the midwives. The researchers were flexible to work around the availability of the midwives. Labour wards are very busy; thus at times, the researchers had to wait for midwives to complete the duties first and hand over the women to other midwives while doing the interview. Most of the interviews were done at the end of the day shift at 19:00 after staff changes, due to the hectic and busy periods of the labour ward.

Informed consent was obtained before conducting the interview. Each interview lasted between 30 and 45 min duration. The interviews were held in English. The researchers used the semi-structured interview guide to aid the interview. Probing and clarification were performed to gain a full understanding of the comments and responses during the interview. Throughout the data collection, the researchers kept a journal to write field notes. Also, an audio recorder was used once consent was gained from participants to record the interviews. The interviews were ended once data saturation was reached when no new information was emerging from the interviews.

### Data analysis

The data analysis followed eight steps of the Tesch analysis method.^20^ This method of data analysis was chosen due to its ability to convert written verbatim transcripts and audiotaped data into a more narrative form.^20^ (1) During the first step, the researchers read the entire transcripts to obtain a sense of the whole and (2) thought about the underlying meaning of the information and (3) then made a list of emerging themes and grouped similar themes together as gathered from the transcripts. (4) The process was repeated with all transcripts and codes assigned to the grouped themes. (5) Furthermore, the researchers found more descriptive wording for the themes to show the relation of the identified themes and sub-themes. (6 and 7) Final arrangement made and preliminary analysis was made by asking peer researchers to read the transcripts to confirm the themes gathered. (8) Consensus was reached between the researchers and the peer researchers.

### Trustworthiness and rigour

Measures of trustworthiness refer to ensuring the truth value of the research findings by Guba and Lincoln (1985 under reference 21 of Polit and Beck). The researchers ensured truthfulness through authenticity, credibility, confirmability and transferability.^21^ First, credibility was ensured through prolonged engagement, where the researchers stayed for a long duration of four months in the data collection field and data were triangulated using various data collection sources. Confirmability was ensured firstly through member checking at the end of interview; the researchers gave a summary of the interview and an audit trail performed where the participant was asked to verify whether all the points were captured accurately. Furthermore, both the researcher and co-researcher verified all the themes and sub-themes generated from the data collected. Furthermore, transferability was ensured by recording the entire interview using the audiotapes and conducting thick description was ensured through the extensive literature review of the phenomenon studied.

### Ethical considerations

Ethical clearance to conduct the study was granted by the University of Pretoria, Research Ethics Committee, Faculty of Health Sciences before commencement of the actual research (ethical clearance certificate: 133/2018). Permission was obtained from the Department of Health and the Chief Executive Officer of the hospital in Tshwane.

The researchers maintained the following ethical principles that guided the study: Beneficence, respect for human dignity and justice as outlined in the Belmont report. Informed consent was sought from the participants before the data collection commenced. Participants were informed of their right to partake or withdraw from the study at any given point. Participants were informed that should they express any discomfort during the interviews; additional psychological support would be made available to them.

## Findings

The analysis process yielded the following three themes as indicated in Table 2.

**TABLE 2:** Themes and sub-themes that emanated from results.

Themes	Sub-themes
Theme 1: Barriers to culturally appropriate care in maternity service	Sub-theme 1.1: Ethno-linguistic barriers
	Sub-theme 1.2: Uncooperative behaviour of women during birth
Theme 2: Midwives imposing own personal adapted cultural beliefs on childbearing women	Sub-theme 2.1: Midwives’ own adopted culture
	Sub-theme 2.2: Disrespect of tradition and culture by midwives
	Sub-theme 2.3: Midwives’ preconceived fears regarding childbirth
Theme 3: Midwifery Unit workplace culture	Sub-theme 3.1: ‘Following what the Roman’s do in Rome’

### Theme 1: Barriers to culturally appropriate care in maternity care service

The barriers to culturally appropriate care in maternity services have been identified as the main theme. The midwives in the study indicated that it is challenging for them to care for women from various ethnic groups. The following sub-themes also emanated from the main theme: ethno-linguistic barriers and due to not being able to understand the childbearing women language and cultural beliefs of the following women for example (women from Somalia or women from Zimbabwe speaking Shona), these are unfamiliar languages to the midwives. The consequence of ethno-linguistic barrier becomes and results in uncooperative behaviour of the women during labour also becomes a barrier to provide the cultural appropriate care. The following quotes support the main emergent theme.

#### Sub-theme 1.1: Ethno-linguistic barriers

The midwives in the study indicated that they experience ethno-linguistic difficulties during birth in which they are unable to understand what the women are saying especially when dealing with the various ethnic groups of women who utilise the maternal healthcare services in South Africa. Lack of understanding of these women’s language hampers the midwife’s ability to provide care that is in line with the womens’ cultural preferences regarding birth and maternal positions to adopt during the second stage of labour:

One of the midwives reported on their experience that:

‘I had an experience one time with a Shona women, we could not communicate due to language barrier. This woman was also not following instructions given. It’s easy for the midwife to use lithotomy/supine position because; this woman was wrestles and I could not convince her to use other birth position such as squatting …’ (Participant 5, registered nurse/midwife, 32 years old)

Another midwife indicated that they even try to use a translator or non-verbal gestures when dealing with women who do not understand English or their native languages:

‘We get a lot of women coming from other African countries who don’t understand English to deliver at our unit. You cannot instruct someone who does not understand you. So with lithotomy position it’s safe because, once they look up maybe you the midwife can look at a woman during birth and use gestures to instruct her. With other birth positions like squatting, they might be looking down and not understanding what you are saying.’ (Participant 11, registered nurse/midwife, 31 years old)

Another view regarding women’s culture came out of the interviews:

‘We deal with women from East Africa who come with those traditional herbs mixed in bottle. We cannot allow that in the ward and others even tell you that from my culture I am used to squatting because that is how I have been taught. We as midwives we cannot allow that.’ (Participant 4, registered midwife, 24 years old)

Notably, the issue of language and communication difficulty came as a barrier for midwives to provide culturally appropriate care.

#### Sub-theme 1.2: Uncooperative behaviour of women during birth

The second sub-theme that emanated from the findings was the uncooperative behaviour of women during birth, experienced as a result of a language barrier with the women from different ethnic groups. The midwives in the study told that most of the time women do not cooperate during labour. Lack of cooperation makes it difficult for them to try and think of utilising other maternal birth positions. The quotes below support the sub-theme identified.

One of the midwives indicated that:

‘The challenges we are facing as midwives have to deal with uncooperative patients, especially from the women from other countries, and sometimes with the increased workload and high patient influx we are not able to use other birth positions …’ (Participant 6, registered nurse/midwife, 29 years old)

Another midwife raised an assumption that childbearing women have cultural beliefs and practices and have their own preferences during labour, but midwives are not able to cater for the cultural preferences. The language barrier meant that there was a lack of understanding between midwives and childbearing women:

‘The women will tell you that in their culture, they prefer to squat. Most of these women from African countries are uncooperative patients who fear childbirth pain; they start lifting buttock during labour, while of lithotomy position. The others even close their legs during labour, but it is a better position because you can see the perineum, how will they be able to co-operate on position such as squatting.’ (Participant 11, registered nurse/midwife, 31 years old)

It is noted that midwives are not rendering culturally appropriate care due to the challenges resulting from the lack of cooperation from most of the women from other ethnic groups because of the language barrier.

### Theme 2: Midwives imposing own adopted cultural beliefs on childbearing women

The findings of the study identified the second main theme, which indicates that midwives impose their own cultural attributes on childbearing women. The midwives in this study disregarded the childbearing women’s cultural preference concerning the maternal position. The following sub-themes emerged to support the main theme including the conundrum of own personal cultural beliefs and midwives’ preconceived ideas and fears regarding labour.

#### Sub-theme 2.1: The midwives’ own adopted culture

This sub-theme describes midwives’ adopted culture (*culture explained as a way of doing things*), which poses a challenge to them taking into consideration women’s preferences and cultural beliefs concerning maternal positions during birth. One of the midwives indicated that they normally follow their own personal beliefs:

‘I personally prefer using the supine position, as it is a position I prefer. Moreover, I developed the culture and norm for using the lithotomy position, It is a position I prefer irrespective of what the women prefers of chooses …’ (Participant 6, registered nurse/midwife, 29 years old)

The midwives acquired this norm in the wards where they practised their midwifery skills. They learned the cultural practice and followed the practice done in the ward:

‘The lithotomy position is a position most of us are using somehow it’s like a norm in the ward. We have never tried other positions, I mean everywhere I worked before as a student it’s a position that is always used …’ (Participant 3, registered nurse/midwife, 30 years old)

Midwives follow their own norms and disregard the women’s norms:

‘The other factor that contributes to midwives not using other birth positions is because of comfortability. You know the religious position the lithotomy position is mostly done for the comfort of midwife, they’re not much concerned of patients comfort but the midwife needs to be more comfortable with position …’ (Participant 20, advanced midwife, 40 years old)

It is evident from the findings that midwives found an existing culture in the maternity wards and adopted the culture of following the norm in the ward and disregard the cultural beliefs that women have concerning their childbirth process.

#### Sub-theme 2.2: Disrespect of women’s tradition and culture by midwives

The participants reported disrespect or disregard for the traditions and culture of women who come to give birth. They acknowledged that they do not recognise the traditions and norms but instead encourage the westernised, evidence-based medicine that is provided in the hospital. The following quotes support the sub-theme.

One of the midwives told that women would indicate that they prefer to squat in their own preferred culture:

‘The women will tell you that in their culture, they prefer to squat [*Shona women from Zimbabwe*].’ (Participant 11, registered nurse/midwife, 31 years old)

Some midwives would disregard the women’s choices and beliefs:

‘We always found the lithotomy being used here, and no we never give the woman a choice of birth position.’ (Participant 1, advanced midwife, 36 years old)

‘We only use the lithotomy position because we are used to this position …’ (Participant 13 advance midwife, 60 years old)

The midwives further mistreat the women, by indicating that some women are not well educated. This indicates that some women are not treated equally due to their educational background:

‘The women are not literate enough to know their rights or maybe the information that needs to be given to them. To be honest we don’t even inform them at all of the birth positions available, they are not given an option because we are not going to go with her option. We only use birth positions that suit us not the patient …’ (Participant 1, advanced midwife, 36 years old)

#### Sub-theme 2.3: Midwives’ preconceived fears regarding childbirth

The third sub-theme to support the main theme indicates that midwives have preconceived fears concerning labour and that affects them being able to support the women’s choices and cultural beliefs concerning birth. Some of the midwives verbalised their cultural beliefs, and some of the preconceived fears have a role to play on them not allowing the women’s own choice of birth position and not rendering culturally appropriate care to support the women’s choices.

One midwife indicated that the fears she have are a barrier to her providing culturally appropriate care and to give preference to the women’s choice of maternal position:

‘I think we fear of the unknown of what if something goes wrong, we need to break the culture of putting woman on one position …’ (Participant 9, registered nurse/midwife, 24 years old)

Another midwife indicated that other fears included unfamiliarity with other birth positions:

‘Suppose the woman is giving birth on the commode [*birth stool*] and something happens it is not easy to get down there. The woman might not get up quickly, you might not identify the problem quickly for example cord around the neck, and you could end up in a lot of trouble …’ (Participant 7, advanced midwife, 55 years old)

One of the midwives was worried about safety as well:

‘The lithotomy position is safe, isn’t that we want a happy mother and an alive baby at the end of the day. So you have to weigh the options do you want to deliver this woman looking down and not understanding you. Not knowing what will happen? I am unsure about the other positions …’ (Participant 11, registered nurse/midwife, 31 years old)

Finally, one of the other midwives indicated the necessity of receiving training to alleviate the fears as one of the suggestions for them to support the women’s choices:

‘Well it can start with alleviating the fear midwives have of being resistant to change and receive more education, skills and training on other birth positions showing them that there is actually no harm of using alternative birth positions on the mother and the baby and the culture associated to women preferences …’ (Participant16, registered nurse/midwife, 30 years old).

### Theme 3: Workplace culture in the midwifery unit

The last theme that contributes to the midwives’ lack of culturally appropriate care is the workplace culture followed in the maternity unit. This is a culture already followed in the ward, irrespective of the women’s preferences or cultural beliefs concerning maternal positions during the second stage of labour. Institutional norms and practices were reported as barriers that limited the midwives from promoting culturally appropriate care or involving the women in the decision-making processes of their labour. Sub-theme 3.1 supports the main theme as identified.

#### Sub-theme 3.1: ‘Following what the Romans do in Rome’

This sub-theme emanated from the findings from most of the midwives, which indicated that they are following the workplace culture already found in the maternity unit.

One of the midwives indicated that this is a workplace culture that they have been accustomed to:

‘It is more of a developed culture and norm for us to utilise the lithotomy position, It is a position I prefer …’ (Participant 6, registered nurse/midwife, 29 years old)

‘We don’t see other birth positions being utilized in our working institutions, thus we developed a culture in the ward of using the supine position, despite the preference of the woman …’ (Participant 4, community service/midwife, 24 years old)

‘The other factor that contributes to midwives not using other birth positions is because of comfortability. You know the religious position the lithotomy position is mostly done for the comfort of midwife, they’re not much concerned of patients comfort but the midwife needs to be more comfortable with position …’ (Participant 20, advanced midwife, 40 years old)

Some midwives even gave a phrase to describe the culture they are now being accustomed to in the wards, which is ‘Do what the Romans do’. The culture we adopted from the midwifery unit, as expounded by some of the midwives:

‘I place the woman on lithotomy position because it is what I found being done in the unit. I think it is a culture of this unit and I know I was taught on other birth positions during studies but I have never practice it. I guess we are just joined what the Romans do in Rome, so I adopted the culture …’ (Participant 9, registered nurse/midwife, 24 years old)

Another midwife decided to disregard the theoretical lessons learnt during midwifery training and followed the norm in the labour ward:

‘I only studied other birth positions in school during the third year, in midwifery as an undergraduate. But when you get to practice [*labour ward*] it is like you do what the Romans do, we never got a formal lecture on birth positions. It is like when you go into practice, and find things being done like that and you just follow what they do …’ (Participant 16, registered nurse/midwife, 30 years old)

## Discussion

### Barriers to culturally appropriate care in maternity care service

The purpose of the study was to explore the midwives’ perspectives on culturally appropriate care to support maternal positions during the second stage of labour. The key findings of the study indicate that currently culturally appropriate care is not rendered in the maternity units, due to barriers such as organisational culture and the personal adapted beliefs of the midwives that prevent them to honour the culture of the childbearing women. The study argues that maternal healthcare professionals need to be more aware of the cultures and subcultures of the childbearing women across all ethnic groups and understand the women’s preferences and choices regarding the maternal position in order to abide by the WHO strategy for culturally appropriate care.^4^ Culturally appropriate care can be as simple as respecting the language of the childbearing women, as it was found in this study that the language barrier was actually an obstacle to the provision of culturally appropriate care, which is not in line with the WHO strategy. A systematic review argues that the lack of culturally appropriate maternity care services has an effect on the women’s uptake of skilled birth care during pregnancy, birth and postpartum period.^4^ Thus, it is mandatory that SBA (including midwives) should treat and care for all women equally irrespective of the women’s cultural or ethnic backgrounds. The minority of ethno-linguistic groups in most countries have poor access to the maternal healthcare system, thus consequently exposing them during pregnancy and childbirth to poor maternal health outcomes^1^. In this study, it was noted that the midwives need to be aware of the women’s cultural choices from all ethnic groups. The interpersonal interaction between the midwife and pregnant woman should foster respectful maternity care (RMC), which respects women’s choices during labour as mandated by the WHO.^22^ Findings emanating from a systematic review indicate results from countries such as Australia (targeted the Indigenous Aboriginal and Torres islander women); United states indicated that the following interventions to foster culturally appropriate care in maternal health should include training staff members on different cultural practices to improve cultural awareness, using health professionals with the shared linguistic background as the patients (in this regard the childbearing women) and lastly incorporating culturally appropriate practices.^1^ This is very true for South Africa, and these recommendations can be made to maternal health services in order to promote culturally appropriate care.

### Midwives imposing own adopted cultural beliefs on childbearing women

Midwives should not impose their own adopted culture regarding the way of doing things and disregard the women’s choices during labour. It is evident from the midwifery curriculum that the midwives are taught all the various birth positions that can be used. The maternity guidelines in South Africa^7^ further indicate that women should be given the options; however, this is not done within the study context. Studies indicate that the lack of women’s autonomy to make informed decisions regarding their own care, which is seen as mistreatment of women during labour.^23^ High-quality RMC is a global priority. The WHO^2,22^ released eight standard areas of RMC. One of the standards includes respect and commitment; this can be further explained as implementation of women-centred care, and informed decision-making processes should be respected by the midwives.^23^ Furthermore, the WHO quality care standards regarding the protection of human rights in childbirth need to be adhered to, as they are essential to optimising positive birth outcomes.

In countries such as Ethopia,^24^ it is further indicated that women are mistreated at health facilities. This affects their choice to give birth at facilities or even accessing the facilities on time, which may also contribute to some of the avoidable causes of mortality and morbidity rates. Numerous studies show the importance of midwives changing their attitudes and start being culturally sensitive to women. Being culturally appropriate fosters women-centered^25^ care that encapsulates the biological, psychological, social and cultural tenets of care during labour as envisaged by the WHO recommendations on Intrapartum Care for a Positive Childbirth Experience.^22^ The midwives should work in partnership with the women thus improving the women’s experience of childbirth.^24^ Furthermore, the midwife needs to change her mindset, towards humanising childbirth in order to provide culturally appropriate care and to give preference to the women’s choice around the birthing position. Furthermore, it is significant that the midwives respect the tradition of the women if it does not impact the safety and quality of care.^26^

### Workplace culture in the midwifery unit

The last theme identified the workplace culture acting as an obstacle to the provision of culturally appropriate care in midwifery units. Many studies indicate that the midwifery unit should promote humanising caring standards and promote RMC. The current findings of the study are in synergy with the recent systematic review by Bradley.^27^ The systematic review designed a conceptual framework that further explores the different stakeholders that contribute to intrapartum care and the role played regarding maternal health services. From the macro-level factors, things such as policy play a role in the practice of the midwives. The meso-level factors in this instance being the Midwifery Unit, which includes the environment where currently the midwives are conducting the birth position during the second stage of labour. These factors have an influence on the day-to-day practice in the micro-level of the midwives.^27^ With relevance to the study, the midwives found that the supine position was done routinely in the maternity unit, and they decided to follow suit without regarding the evidence-based protocols, which indicate that the supine birth position should be avoided. To move forward with the provision of culturally appropriate midwifery care, there is a need to address all barriers from macro to micro-level.^27^ Furthermore, research should be implemented on policies that foster culturally appropriate care in the maternity units. At micro-level, midwives need to be aware that childbearing women are individuals with cultural preferences and choices regarding their labour, in this regard maternal position during the second stage of labour. The currently held beliefs that choice of birth position should be limited and the ongoing disrespect for the women’s choice and tradition need to be addressed.^11^

In summary, women cognitively evaluate the experience of labour differently. Some women remember their birth experiences in detail for a lifetime. Therefore, their birth experience does affect how they perceive birthing (Simkin 1991, 1992).^28^ The birth experience influences the woman’s narrative of herself as a mother and a human being and has the capacity to affect her sense of self and well-being.^28^ Thus, it is imperative that midwifery practice needs to be functionally embedded within the healthcare system, which includes available, accessible, acceptable (organisation of care), respectful, understanding strengthens resources and take a non-interventional stance (philosophy). Midwives and other healthcare providers need to be interpersonally and culturally appropriate and maintain clarity of roles and responsibilities in their inter-professional relationships with their patients to ensure optimal maternal and neonatal outcomes.^29^

## Recommendations

Global perspectives from the WHO published guidelines and policies on the provision of culturally appropriate care still need to be implemented in clinical settings. Furthermore, changes to incorporate culturally appropriate care need to be carried on and supported from the macro-level hospital management to ensure it is implemented in the micro-level maternity wards. Recommendations can be made to re-align the protocols to ensure that women are given a choice during labour on their preferred maternal position as it is in line with the women’s cultural beliefs and norms (perhaps include language interpreters as well). Bottom-up approach needs to be followed for changes to the current workplace culture of restricting women to assume other birth positions and restricting women’s movement should start at the micro-level so as to influence care and render culturally appropriate care.^27^

At the micro-level, in-service training can be provided on cultural beliefs that women have during labour, and this will raise cultural awareness about women from various ethnic groups who are not familiar to the midwives in South Africa.

Guidelines for education and training on culturally appropriate care need to be implemented from the higher education institutions. Culturally appropriate care and relation to scientific evidence-based midwifery care interventions should be developed for the institution under study. Other institutions may benefit through the dissemination of findings.

Further research can focus on the experiences of women from various ethno-linguistic groups regarding culturally appropriate care.

## Strength and limitations

The study provided a broader understanding and insight into maternal healthcare services provided to women during the first and second stages of labour. The findings of the study also enhance support and encourage midwives to be more aware of their practice and advocate for them to be supportive and provide culturally appropriate care to women.

The limitation of the study is that the findings cannot be generalised because the study took place in a specific district hospital in Tshwane, Gauteng with a specific population of interest. Although the study was conducted only in one hospital, the findings of the study can be transferable to other public hospitals within and outside the district.

## Conclusion

The findings of this study demonstrate that midwives need to be aware and provide care that considers the childbearing women’s cultural preferences and practices regarding maternal position during labour and ensure the provision of culturally appropriate care to achieve optimal maternal and neonatal health as recommended by the WHO recommendation. Currently, the maternal healthcare is not tailored towards culturally appropriate care and that affects the women’s utilisation of skilled birth facilities.
